# Selection tests and their predictive value in university nursing students’ success in the first year of study

**DOI:** 10.1186/s12909-023-04140-4

**Published:** 2023-03-22

**Authors:** Judith Bruce, Sfiso Emmanuel Mabizela, Amme Mardulate Tshabalala

**Affiliations:** 1 School of Therapeutic Sciences, Faculty of Health Sciences, University of the Witwatersrand, Johannesburg, South Africa; 2 Centre for Health Science Education, Faculty of Health Sciences, University of the Witwatersrand, Johannesburg, South Africa; 3 Department of Nursing Education Faculty of Health Sciences, University of the Witwatersrand, Johannesburg, South Africa

**Keywords:** Academic success, Selection tests, National Benchmark Test, NSC, Nursing students

## Abstract

**Background:**

Academic performance factors, such as school-leaving grades and other academic indicators for selection, play a significant role in student success. The study aimed to determine the best predictors of academic success in the first year of study for nursing studies at a South African university using three National Benchmark Test (NBT) domains and four National Senior Certificate (NSC) subjects.

**Methods:**

We conducted a retrospective review of the admission data of first-time students (n = 317), enrolled on the Bachelor of Nursing between 2012 and 2018. Hierarchical regression was used to explore important variables predicting success in the first year of study. Cross tabulations were used to determine the association between progression outcome, proficiency levels of the NBT and school quintiles.

**Results:**

All predicting variables explained 35% of the variance in the first year of the study. The NBT MAT (Mathematics), Academic literacy (AL), and NSC’s Life Sciences were statistically significant predictors for passing the first year. Analysis of progression outcomes by the NBT proficiency levels suggests that most students begin studies with lower entry-level skills than required, which hinders academic progress. No major differences in academic performance were observed for the students who attended different quintiles.

**Conclusion:**

Selection test results predict areas where students are likely to encounter difficulties and inform the interventions needed to achieve academic success. There may be serious implications for students admitted with low entry-level skills in variables predicting academic success and they would need tailored academic interventions to improve their grasp of mathematical and biological concepts and their ability to read, think and reason.

**Supplementary Information:**

The online version contains supplementary material available at 10.1186/s12909-023-04140-4.

## Background

Academic success and throughput rates have become essential measures of institutional performance in higher education globally. By extension, degree programmes in higher education institutions such as universities need regular scrutiny of their throughput and output data to enable a comprehensive grasp of the factors that impact these measures. Addressing these factors is crucial for the sustainability of academic programmes and their overall contribution to institutional performance.

Failure to attain academic success, particularly in the first year of study, leads to an early exit from education programmes and considerable losses for the student and the institution. [1, 2] For those students who remain in the education system, it means delayed completion and graduation, and for some, a change in degree or career choice, which has become concerning [3] amid critical nursing shortages. Pressure points for attrition appear to be in the first two years of a four-year Bachelor of Nursing (BN) degree, locally and internationally. [4, 5]

In these years of study, attrition and failure are attributed broadly to academic and non-academic factors. Academic factors include a wide range of cognitive traits and tests that provide some measure of an individual’s ability and/or suitability for a program of study. Academic-cognitive skills are usually assessed in four main dimensions: mathematics, language, natural sciences and reasoning skills, mainly through standardised tests and academic records. [6] Non-academic factors, broadly, include socio-cultural-economic perspectives and institutional factors that affect student adjustment, concentration, motivation and wellness – many of these shown to be crucial success factors. [6, 7]

Although not the focus, social constructs such as motivation and academic self-concept have been shown to have a significant positive association with academic achievement [8, 9], specifically among nursing students [10, 11] and students in distance learning contexts. [12] Academic performance factors such as school-leaving grades, Grade Point Average (GPA) and other scores such as the National Benchmark Test (NBT) for selection play a significant role in student success and retention. In the United States (US), students with higher GPAs in high school and college and higher grades in recently completed nursing courses were more likely to be successful and to remain in the program. [13] Comparable results were also reported at King Saud University, where the high school grade average, aptitude tests and achievement test were found to be significantly associated with allied health students’ academic performance. [14]

In the context of South Africa’s divisive past along racial lines, many institutions of higher learning have restructured their admission policies to redress inequality and injustices associated with it [15]. Amid other changes, this involved the introduction of new metrics to diversify and improve access to university education in general and nursing in particular. The study institution selects applicants for nursing studies based on NBT scores and high school grades or National Senior Certificate (NSC). These selection criteria are exclusively used to determine an admission score - their value in predicting nursing students’ ability to succeed is poorly understood. Previous studies, collated in a scoping review, have shown that cognitive ability has high predictive value for success, hence its continued use in nursing education systems [16], in more equal societies. Evidence from our study would inform decisions about the continued use of these tests as the mainstay of BN selection.

In this study, we aimed to determine the best predictors of academic success based on the selection tests (NBT and NSC), and the association between first-year progression outcome and NBT proficiency levels and school quintiles. We were interested in the proportion of variance explained by the NBT domains and NSC subjects in predicting students’ first-year academic success and identifying the best predictors for passing the first year of study.

With the focus of this paper on cognitive factors only, academic success in the context of the baccalaureate program was defined as the award of a pass mark (≥ 50%) for all first-year courses, permitting a student to proceed to the second year (progression outcome). The study is based on the premise that if we better understand why students fail in the first year despite being selected on academic merit, recommendations for support and/or policy changes can be directed at selection processes that concentrate on cognitive factors only.

### South african schooling and higher education system

In an attempt to redress past inequalities in education, South African schools are classified into five quintiles based on the unemployment rate and literacy level of the community where the school is located; poor communities are home to quintile 1 schools (SQ1) whereas quintile 5 schools (SQ5) are found in more affluent communities. [17] This ranking aims to improve government funding to impoverished schools provided in the National Norms and Standards for School Funding. [17, 18] Schools classified as SQ1 and SQ2 are located in impoverished areas and would receive more funding per child, while SQ5 in affluent areas receive comparatively less government funding. [18, 19] More than 20 years on, the school quintile system may have addressed the funding disparities but not the quality of education and applicants’ ability to access university education. Teachers with good subject knowledge, such as in Mathematics, are concentrated in quintile 5 schools [20] which produce more university entrants and graduates. [21] This means that lower quintile schools would continue to produce candidates who are ill-equipped for university studies.

On completing high school, school-leavers are awarded a National Senior Certificate (NSC) that describes and categorises school performance and suggests the higher education program the students may qualify for i.e. Higher Certificate, National Diploma or Bachelor’s degree. The National Benchmark Tests (NBT) and the NSC are generally used to select students for places in the Bachelor of Nursing (BN) program at universities in South Africa. However, some universities use the APS, a score calculated from the NSC subjects. [15] The main difference is that the NSC is an assessment tool for learners exiting high school, whereas the NBT is a tool for entry-level benchmarking into universities. [22] Applicants are ranked using a Composite Index (CI), where the NBT and NSC each contribute 50%. The CI is derived from three NBT domains and five NSC subjects (English, Mathematics, Physical Sciences or Life Sciences, and two subjects in which an applicant attains the highest mark, except for the subject Life Orientation. [23] These two assessments differ in their purpose, design, and intention. [24]

The NBT is a criterion-referenced test designed to assess students’ entry-level skills in three domains: NBT mathematics (NBT MAT), NBT academic literacy (NBT AL) and NBT quantitative literacy (NBT QL). NBT MAT tests prospective students’ understanding of mathematical concepts related to mathematics, physics, and chemistry. NBT QL is designed to assess students’ abilities to engage with mathematics and the quantitative demands of higher education; NBT AL assesses students’ reading skills, reasoning skills, and ability to engage with the language demands of higher education. [23, 25] Performance in these domains is categorised into levels that indicate the entry-level skills and the recommended support the student would need from the university. [23]

## Methods

### Study sample

All BN students on the Business Intelligence Service (BIS) database that were enrolled in the first year of study between 2012 and 2018 were included (n = 317). To determine the predictive validity of the selection tools for passing the first year of study on the initial attempt, we excluded the data of students who failed cancelled and those who did not have complete data for our variables of interest (NBT domains and four NSC subjects). After assessing the data for assumptions, 146 cases were analysed from the pool of 178 students who passed. The entire sample was included to analyse the links between the NBT proficiency levels, school quintiles and students’ progression outcome, indicating whether a student is permitted to proceed (has passed), has failed or cancelled.

### Data collection

We conducted a retrospective review of secondary data obtained in 2020 from the (BIS), an analytics, and research unit of the University. The BIS collects students’ pre-admission data and collates progression outcomes by year of study. Data sets are available at the request of researchers in the university, subject to ethical approval. The requested demographic data included variables such as gender, race, and place of origin and school quintile. The requested academic variables were NBT results for all three domains, four NSC subjects, and first-year academic performance.

### Data analysis

The data were analysed using IBM SPSS V27 and assessed for the hierarchical regression assumptions (normality, linearity, inter-correlations, homoscedasticity, and Mahalanobis distance). [26–29] We employed two regression models that enabled their *R*^2^ coefficients to be compared. In the first model, only the NBT domains (NBT MAT, NBT AL, and NBT QL) were entered in the regression equation, and in the second model, the four NSC subjects (English, Mathematics, Physical Sciences, and Life Sciences) were added as independent variables. The dependent variable was the average of first-year courses. This made it possible to compare and report the *R*^2^ coefficients of the NBT domains, the *R*^2^ change after adding the NSC subjects and the combination of variance explained by all predicting variables [24]. Unstandardised (*B*) and standardised (β) regression coefficient levels for each variable were examined and reported. ANOVA *F* statistic was used to confirm the predictive strength of the model, and *R*^*2*^ coefficients were used to calculate the effect size. Cross-tabulation tests were used to determine the links between progression outcome and the NBT domains and school quintiles.

### Ethics approval and consent to participate

The need to obtain informed consent from the students was waived by the Human Research Ethics Committee (Medical) (HREC). The HREC is an independent committee at the University of the Witwatersrand, which is registered with the National Health Research Ethics Council (NHREC) of the South African Department of Health. The HREC granted approval to conduct the study and the ethics number is M220561. Secondary data were used in this study and de-identified appropriately to ensure anonymity.

## Results

### Sample demographics

We analysed the admission data of 317 BN students registered for the first time between 2012 and 2018. The highest proportion of students was categorised as African (80%) and the lowest as Indian (3%). There were more female (83%) than male students (17%); the majority were from urban areas (79%) and attended well-resourced SQ4 and SQ5 schools (65%). In terms of progression outcome, 56% of students (n = 178) passed the first year of study, 33% (n = 104) failed, and 11% (n = 35) discontinued their studies during the first year. See Table 1 below for a detailed description of the sample.

**Table 1:** Sample demographics (n = 317)

Variable	Category	Frequency	%
Cohorts	2012	32	10%
	2013	39	12%
	2014	46	15%
	2015	47	15%
	2016	55	17%
	2017	46	15%
	2018	52	16%
Race	African	253	80%
	White	40	13%
	Coloured	13	4%
	Indian	11	3%
Gender	Male	53	17%
	Female	264	83%
Place of origin	Rural	54	17%
	Urban	251	79%
	Unknown	12	4%
School Quintile	SQ1	18	6%
	SQ2	31	10%
	SQ3	49	15%
	SQ4	49	15%
	SQ5	157	50%
	Unknown	13	4%
Progression outcome	PCD	178	56%
	Failed	104	33%
	Cancelled	35	11%

### NBT performance levels

Most students were placed at an Intermediate Lower level for NBT MAT (n = 179; 56.5%) and for NBT QL (n = 163; 51.4%). Results for NBT AL show that about 46% (n = 145) were at an Intermediate Upper level. More than a quarter of the students (27%) were Proficient in all three NBT domains, with under 3% proficient in NBT MA and NBT QL, as shown in (Table 2).

**Table 2:** Frequency distribution of performance levels (n = 317)

NBT performance levels	NBTMA	NBTAL	NBTQL
Proficient	4 (1.3%)	73 (23%)	9 (2.8%)
Intermediate Upper	25 (7.9%)	145 (45.7%)	73 (23%)
Intermediate Lower	179 (56.5%)	87 (27.4%)	163 (51.4%)
Basic	92 (29%)	9 (2.8%)	69 (22.8%)
Missing	17 (5.4%)	3 (0.9%)	3 (0.9%)

### Variance explained by NBT and NSC (n = 146)

In model 1, the NBT domains explained 18% of the variance, which was statistically significant (*R*^2^ = 0.181, *F* (3, 142) = 10.493, *p* < .000). When Life Sciences, English, Mathematics and Physical Sciences were added (model 2), the NSC subjects accounted for 17% of the variance (*R*^2^ = 0.175, *F* (7, 138) = 9. 381, *p* < .000. When all predicting variables were added in the regression equation, they accounted for 35% of the variance (*R*^2^ = 0.356, *F* (7, 138) = 10. 919, *p* < .000). In the first model, NBT Mathematics, NBT Academic Literacy were significant predictors of success. In the second model, where all variables were combined, NBT MAT, NBT AL and the NSC subject, Life Sciences, were significant predictors of success in the first year of the BN program; the *R*^*2*^ coefficient departed significantly from zero, confirming the model’s predictive utility. The effect size of the whole model was large (*f*^2^ = 0.55). Refer to Table 3 for unstandardised (*B*) and standardised (β) regression coefficients for all variables.

**Table 3:** Contribution of predicting variables to success in the first year of study (n = 146)

	Variables	B [95% CI]	Beta	Sig
Model 1	NBT Mathematics	0.176 [0.094, 0.258]	0.333	0.000*
	NBT Academic Literacy	0.182 [0.102, 0.262]	0.362	0.000*
	NBT Quantitative Literacy	− 0.061 [-0.137, 0.015]	− 0.128	0.112
Model 2	NBT Mathematics	0.116 [0.035, 0.197]	0.220	0.005*
	NBT Academic Literacy	0.173 [0.094, 0.253]	0.345	0.000*
	NBT Quantitative Literacy	− 0.055 [-0.125, 0.016]	− 0.114	0.127
	English	0.092 [-0.013, 0.197]	0.133	0.087
	Mathematics	0.004 [-0.103, 0.112]	0.009	0.938
	Life Sciences	0.150 [0.041, 0.259]	0.236	0.008*
	Physical Sciences	0.076 [-0.014, 0.166]	0.178	0.097

### NBT, school quintile and first-year progress outcome n = 317

**Fig. 1:**
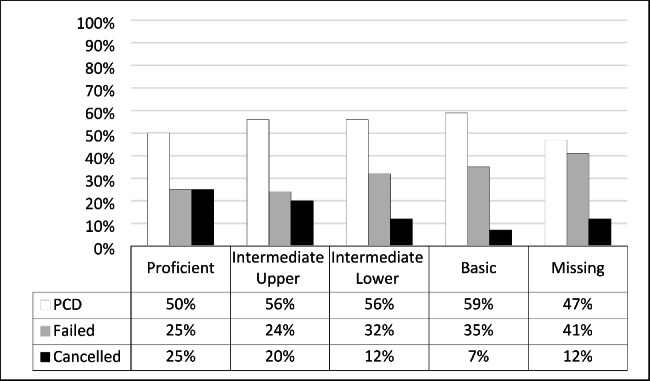
NBT Mathematics and first-year progression outcome (n = 317)

Students’ progression outcome for NBT MAT in the Intermediate Lower performance level shows that 56% (n = 100) passed and 32% (n = 58) failed; 12% (n = 21) cancelled their studies. For students ranked at a Basic level of performance, 59% (n = 54) passed, and 35% (n = 32) failed; the remaining 7% (n = 6) cancelled (Fig. 1).

**Fig. 2:**
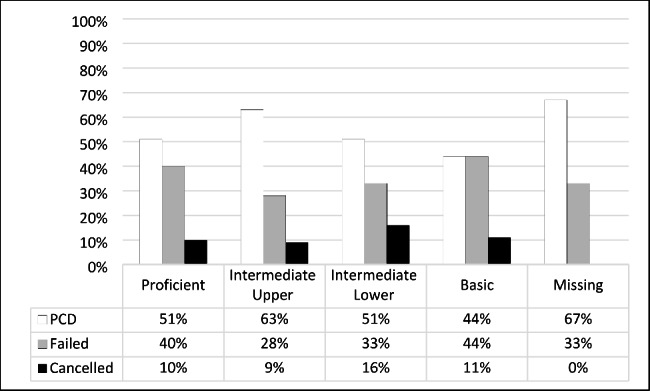
NBT Academic literacy and first-year progression outcome (n = 317)

Of the students rated as Proficient in NBT AL, 51% (n = 37) received a pass progression outcome, 40% (n = 29) failed, and 10% (n = 7) cancelled their studies. In the Intermediate Upper level, 62.7% (n = 91) passed, 28.3% (n = 41) failed, and 9% (n = 13) cancelled their studies. Of the 87 students in the intermediate lower level, 51% (n = 44) passed, 33% (n = 29) failed, and 16% (n = 14) discontinued (Fig. 2).

**Fig. 3:**
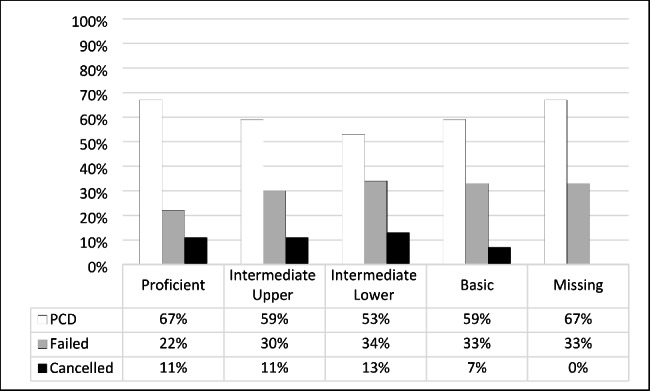
NBT Quantitative literacy and first-year progression outcome (n = 317)

An analysis of students’ progression outcome for NBT QL in the Intermediate Upper level shows that 59% (n = 43) passed and 30% (n = 22) failed, and 11% (n = 8) cancelled. Of the students in the Intermediate Lower level, 53% (n = 86) passed, and 34% (n = 56) failed; 13% (n = 21) cancelled their studies. Of those at the Basic performance level, 59% (n = 41) passed and 33% (n = 23) failed; 7% (n = 5) cancelled their studies (Fig. 3).

### School quintile and progression outcome

**Fig. 4:**
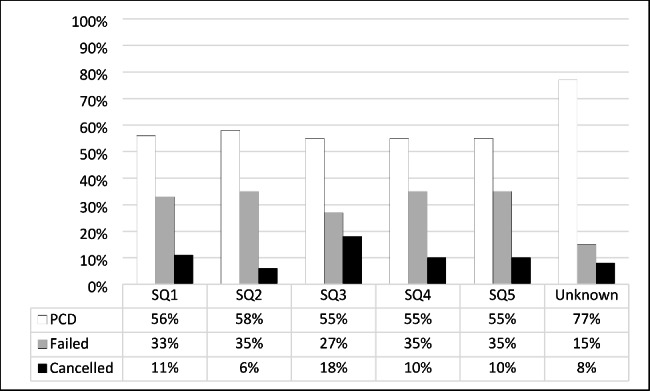
School quintiles and first-year progression outcome (n = 317)

Close to two-thirds of the sample (64.9%; n = 206) attended well-resourced schools (SQ4&5); those from SQ5 achieved a 55% pass rate (n = 86) in the first year, and 35% (n = 55) failed; the remaining10% (n = 16) cancelled their enrolment. Of those who attended SQ4 schools, 55% (n = 27) passed and 35% (n = 17) failed; 15% (n = 49) attended SQ3, the so-called middle schools, of which 55% (n = 27) passed, 27% (n = 13) failed and 18% (n = 9) cancelled. Of the students who attended SQ2 schools (n = 31), 58% (n = 18) passed, 35% (n = 11) failed, and 6% (n = 2) cancelled, and at SQ1 schools (n = 18), 56% (n = 10) passed, 33% (n = 6) failed, and 11 (n = 2) cancelled. Refer to Fig. 4 below for a description of students’ performance by school quintiles.

## Discussion

The sample represented an urbanised majority from mostly well-resourced schools, which falls short of the admission categories intended to redress and diversify the student body. Females were in the majority, which is in keeping with most universities’ student profiles, country-wide showing an annual growth rate of 3.5% and a proportion of 58.5% in 2017. [30]

More than half of the sample (56%) passed the first-year examination, while 11% cancelled their registration. Reasons for discontinuing university studies were not obtainable from the database. This study’s attrition rate (including failure and deregistration) may be an alarming result, but this finding is no different from locally reported attrition rates of between 39 and 58% in the first year of nursing studies. [5, 31, 32] In other health disciplines, such as Physiotherapy, that employ the same selection criteria in the university where this study was conducted, a failure rate of less than 20% is reported. [23] In the US, the National League for Nursing estimates the national attrition rate for nursing programmes to be 20% and as high as 50% in some baccalaureate nursing programmes in the US [4, 33], with most students dropping out in the first semester. More recent writings show that, although there are emerging factors such as place of origin and residence, academic factors are a constant predictor of success for health science students. [23] These factors would need further investigation in our study context.

### Predictors for passing the first year

For successful students, the NBT domains accounted for 18%, and the NSC subjects explained 17% of the variance in passing the first year of study. All predicting variables explained 35% of the variance in passing the first year of study. The results show that aspects of the NBT and NSC are valuable predictors of performance in the first year of study and based on the effect size (*f*^2^ = 0.55), there is confidence in the selection tests as the best predictors of academic success. This is similar to findings of local studies of economics and physiotherapy students that, on average, both NBT and NSC results are helpful predictors of performance in first-year of study. [23, 34]

Of the NSC subjects required for nursing studies, Life Sciences’ performance was a significant predictor of academic success in the first year of nursing (p < .005). Local scholars also reported similar findings for the subjects Life Sciences and Mathematics. [35, 36] However, in this study, NSC mathematics was found not to be a significant predictor for passing the first year (p > .005). In both regression models, the mathematics and academic literacy components of the NBT were found to be significant predictors for passing the first year of study. This finding underscores the importance of students comprehending mathematical concepts in subjects such as physics and chemistry and their integration into nursing science, including the academic writing and language demands for developing reasoning and critical thinking skills.

Performance in the NBT shows that the entry-level skills of nursing students are comparatively low despite the majority (70%) having received their education at well-resourced schools and having been selected on academic merit. Less than half (46%) performed at the Intermediate Upper level in academic literacy, with a low NBT performance (Basic and Intermediate bands) for the majority of students in NBT MAT (85.5%) and NBT QL (74.2%). These performance findings match those of the Centre for Educational Testing for Access and Placement (CETAP) [25] in that nearly 90% of students in these bands would need extensive support in mathematics and quantitative literacy.

While entry-level NBT performance has significant importance, academic literacy skills should be constantly developed beyond the first year to ensure that these skills become embedded in students’ character as emerging nursing professionals. In this regard, findings of a systematic review suggest using discipline-specific literacy genres for developing critical thinking and reasoning specific to nursing. [37] Course interventions to strengthen academic literacy in Humanities students show an upward trajectory from their baseline performance. [38] Most first-year students fall in the Intermediate Upper and Lower performance bands for academic literacy, so they would need complementary academic support, including language-intensive activities, designated courses, tutorials and the like. Those students at the Basic NBT performance level would possibly not cope with the demands of the degree and add to the attrition rate. Where students are admitted at this level as part of transformation imperatives, the institution must provide dedicated academic support systems and resources; this may include foundation and extended degree programmes. [23, 38, 39] Among Physiotherapy students, it was found that a low score in NBT AL might result in a poor performance outcome due to academic language demands as well as poor reading and reasoning ability [40], whilst weaknesses in quantitative literacy affect students’ ability to transfer quantitative ideas from learning contexts to workplace contexts. Mutakwa and Mhakure [38] confirm the benefits of using the NBT QL as a valuable indicator of numerical mastery of students coming into university as well as an indicator of the academic interventions needed in the form of extended degree programmes.

### School quintile and progression outcome

The percentage pass of the sample was almost evenly split between well- and poorly-resourced schools. Schools in lower quintiles are presumed to achieve far less than those in higher quintiles. [17] However, in our study, although not comparative of the outcomes in quintile location, 57% of SQ1 and SQ2 students passed the first year compared to 55% of SQ4 and SQ5 students. Although the numbers are smaller, this result points to the potential for success of nursing applicants from poorly resourced schools. A further explanation from a local study by van Dyk & White [17] suggests that the current quintile ranking system is not flawless and might allow for inaccurate ranking of Schools. The school quintile may not be useful in predicting academic success and is unrelated to first-year progression outcomes for nursing students.

## Limitations

There are many variables linked with students’ academic performance. The admission categories to select students were not explored to understand other differences in students’ performance. Categories with low expected counts were not merged, but the results were reported. These limitations should be considered to understand students’ entry-level skills and chances of success comprehensively.

## Conclusions and recommendations

Determining whether selection tests predict success in the first year of nursing studies was the study’s main endpoint. Mathematics and academic literacy components of the NBT and NSC Life Sciences are significant predictors of success for first-year BN students. Having low entry-level skills in these components means that most nursing students would need tailored academic interventions to improve their grasp of mathematical and biological concepts and their ability to read, think and reason; an extended curriculum or bridging course would be a suitable mode for such interventions, balanced against the resources required. Not all students who were rated as Proficient in all domains passed, which points to the likelihood of other non-academic factors at play. Similarly, there is a large proportion of variance in performance that could be explained by career choice, motivation levels and other personal or social constructs beyond this study’s scope. These factors and the value of school quintiles in selecting nursing students need further investigation.

## Electronic supplementary material

Below is the link to the electronic supplementary material.

Regression results

## Data Availability

The data used in this study are available from the corresponding author subject to ethical approval.

## Declarations

### Disclaimer

The views and opinions expressed in this article are those of the authors and not the university’s official position.

## Abbreviations

NBT: National Benchmark Test
NBTMA: National Benchmark Test Mathematics
NBTAL: National Benchmark Test Academic Literacy
NBTQL: National Benchmark Test Quantitative Literacy
NSC: National Senior Certificate
SQ: School Quintile
