# Supplementary material for: Selection tests and their predictive value in university nursing students’ success in the first year of study
Source: BMC Med Educ. 2023 Mar 22;23:176. doi: 10.1186/s12909-023-04140-4 (PMC10035252; doi:10.1186/s12909-023-04140-4)
Supplement: Supplementary file 1 — Regression results [file 12909_2023_4140_MOESM1_ESM.docx]

Regression results

The figure A1 below shows the Box plot for all variables used in the regression models. As shown in the figure below, there was a presence of mild outliers denoted with a circle. These were considered mild and were not removed from the data set. The assumption of normality was confirmed by virtually assessing residual statistics which indicated that a critical chi-square value for Mahalanobis distance was not exceeded in relation to the number of independent variables used. See the residual Table A1 below.


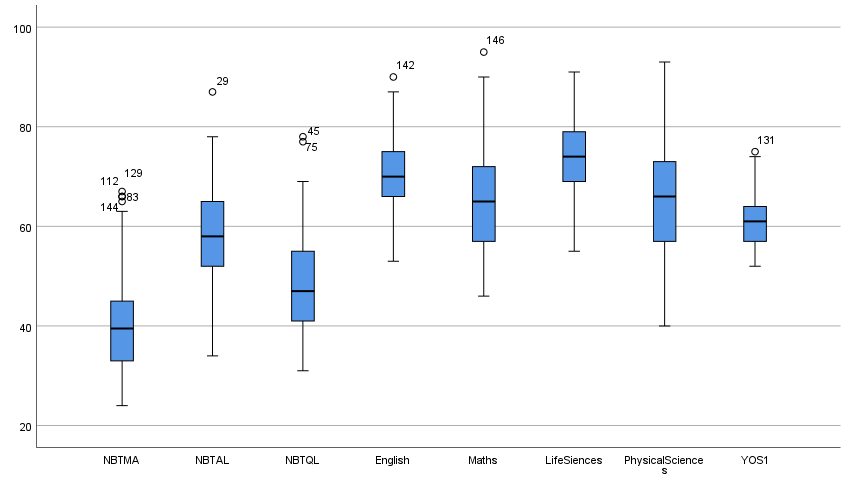


Figure A1: Boxplot for all variables

Table A1. Residuals Statistics

| **Residuals Statistics** | | | | | |
| --- | --- | --- | --- | --- | --- |
|  | Minimum | Maximum | Mean | Std. Deviation | N |
| Predicted Value | 54.69 | 68.58 | 61.08 | 2.859 | 146 |
| Std. Predicted Value | -2.234 | 2.627 | .000 | 1.000 | 146 |
| Standard Error of Predicted Value | .486 | 1.603 | .896 | .215 | 146 |
| Adjusted Predicted Value | 54.52 | 69.10 | 61.07 | 2.900 | 146 |
| Residual | -9.437 | 10.692 | .000 | 3.841 | 146 |
| Std. Residual | -2.397 | 2.715 | .000 | .976 | 146 |
| Stud. Residual | -2.575 | 2.793 | .000 | 1.005 | 146 |
| Deleted Residual | -10.894 | 11.312 | .003 | 4.077 | 146 |
| Stud. Deleted Residual | -2.630 | 2.865 | .001 | 1.012 | 146 |
| **Mahal. Distance** | **1.215** | **23.046** | **6.952** | **3.819** | **146** |
| Cook's Distance | .000 | .128 | .008 | .015 | 146 |
| Centered Leverage Value | .008 | .159 | .048 | .026 | 146 |
| a. Dependent Variable: YOS1 | | | | | |

The assumption of normally distributed residuals was tested further with the Normal probability plot of regression standardised residual. As shown in Figure A2 below, the points are clustered along the diagonal line indicating that residuals are normally distributed.


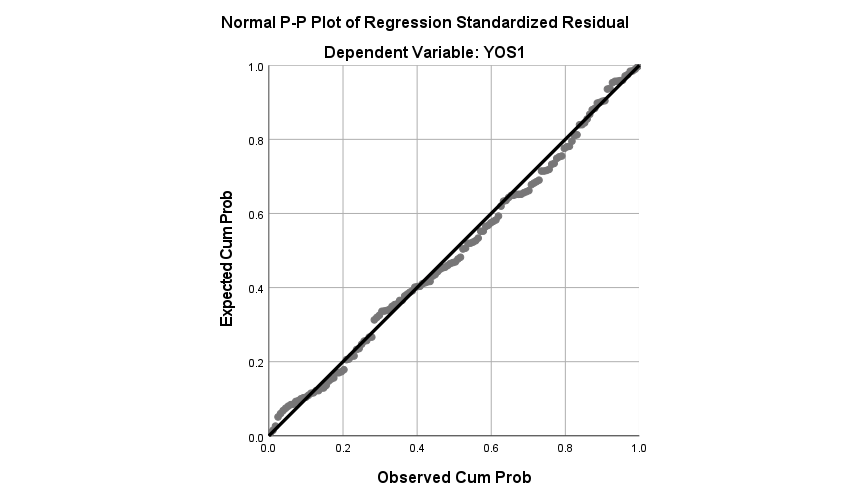


Figure A2: Normal probability plots

The assumption of normality, linearity, and homoscedasticity was not violated. As demonstrated in Figure A3, the scatterplot of standardised residuals against standardised predicted values below indicated an even spread of points which confirms that the assumption was not violated.


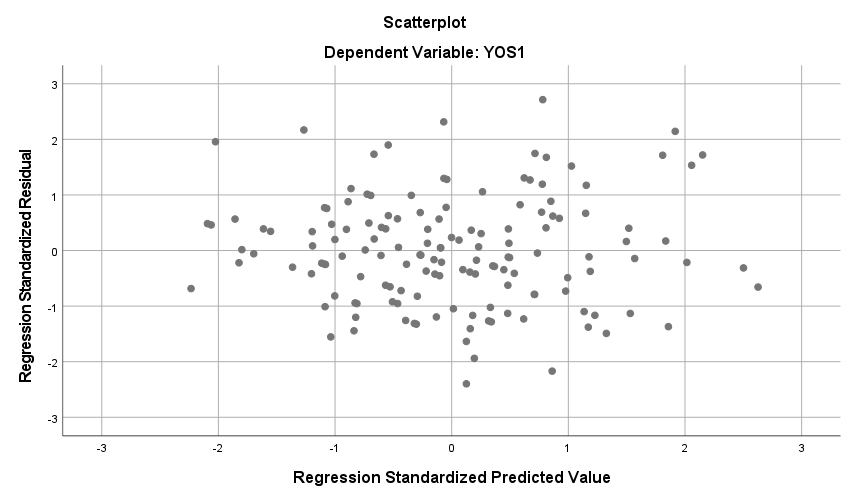


Figure A3: Scatterplot

The Table A2 below shows the *r*^2^ coefficient for two blocks as reported on lines 242-252. In addition, the *r^2^* change is shown when the other set of variables were added in the model is shown.

Table A2: Model summary

| **Model Summary^c^** | | | | | | | | | |
| --- | --- | --- | --- | --- | --- | --- | --- | --- | --- |
| Model | R | R Square | Adjusted R Square | Std. Error of the Estimate | Change Statistics | | | | |
|  |  |  |  |  | R Square Change | F Change | df1 | df2 | Sig. F Change |
| 1 | .426^a^ | **.181** | .164 | 4.377 | .181 | 10.493 | 3 | 142 | .000 |
| 2 | .597^b^ | **.356** | .324 | 3.937 | **.175** | 9.381 | 4 | 138 | .000 |
| a. Predictors: (Constant), NBTQL, NBTMA, NBTAL | | | | | | | | | |
| b. Predictors: (Constant), NBTQL, NBTMA, NBTAL, English, LifeSiences, PhysicalSciences, Maths | | | | | | | | | |
| c. Dependent Variable: YOS1 | | | | | | | | | |

The ANOVA test in Table A3 below shows the model's predictive utility and that the *r^2^* coefficient departs significantly from zero. From the *r*^2^ change of this model, we can assume that the predicting variables explained a statistically significant variance in the outcome variable.

Table A3: ANOVA

| **ANOVA^a^** | | | | | | |
| --- | --- | --- | --- | --- | --- | --- |
| Model | | Sum of Squares | df | Mean Square | F | Sig. |
| 1 | Regression | 603.176 | 3 | 201.059 | 10.493 | .000^b^ |
|  | Residual | 2720.996 | 142 | 19.162 |  |  |
|  | Total | 3324.171 | 145 |  |  |  |
| 2 | Regression | 1184.854 | 7 | 169.265 | 10.919 | .000^c^ |
|  | Residual | 2139.317 | 138 | 15.502 |  |  |
|  | Total | 3324.171 | 145 |  |  |  |
| a. Dependent Variable: YOS1 | | | | | | |
| b. Predictors: (Constant), NBTQL, NBTMA, NBTAL | | | | | | |
| c. Predictors: (Constant), NBTQL, NBTMA, NBTAL, English, LifeSiences, PhysicalSciences, Maths | | | | | | |

In Table A4 below, the unique contribution of each variable in the model is explained, Futhermore, Table A4 provides information about which variables were significant. The B weights represent the expected change in the criterion as a result of one unity change in the other predictor. The Beta values reflect the anticipated change in the standard deviation in the criterion associated with a 1 SD change in the relevant predictor while taking other predictors into account.

Table A4. Coefficients

| **Coefficients** | | | | | | | | | | | | | | |
| --- | --- | --- | --- | --- | --- | --- | --- | --- | --- | --- | --- | --- | --- | --- |
| Model | | Unstandardised Coefficients | | Standardised Coefficients | t | Sig. | 95,0% Confidence Interval for B | | Correlations | | | Collinearity Statistics | |  |
|  |  | B | Std. Error | Beta |  |  | Lower Bound | Upper Bound | Zero-order | Partial | Part | Tolerance | VIF |  |
| 1 | (Constant) | 46.416 | 3.153 |  | 14.724 | .000 | 40.184 | 52.648 |  |  |  |  |  |  |
|  | NBTMA | .176 | .042 | .333 | 4.238 | .000 | .094 | .258 | .252 | .335 | .322 | .934 | 1.071 |  |
|  | NBTAL | .182 | .040 | .362 | 4.513 | .000 | .102 | .262 | .272 | .354 | .343 | .894 | 1.118 |  |
|  | NBTQL | -.061 | .038 | -.128 | -1.599 | .112 | -.137 | .015 | .008 | -.133 | -.121 | .905 | 1.105 |  |
| 2 | (Constant) | 26.340 | 4.687 |  | 5.619 | .000 | 17.072 | 35.608 |  |  |  |  |  |  |
|  | NBTMA | .116 | .041 | .220 | 2.835 | .005 | .035 | .197 | .252 | .235 | .194 | .777 | 1.287 |  |
|  | NBTAL | .173 | .040 | .345 | 4.300 | .000 | .094 | .253 | .272 | .344 | .294 | .726 | 1.378 |  |
|  | NBTQL | -.055 | .036 | -.114 | -1.537 | .127 | -.125 | .016 | .008 | -.130 | -.105 | .851 | 1.175 |  |
|  | English | .092 | .053 | .133 | 1.724 | .087 | -.013 | .197 | .330 | .145 | .118 | .781 | 1.281 |  |
|  | Maths | .004 | .054 | .009 | .078 | .938 | -.103 | .112 | .219 | .007 | .005 | .392 | 2.552 |  |
|  | LifeSiences | .150 | .055 | .236 | 2.712 | .008 | .041 | .259 | .432 | .225 | .185 | .616 | 1.624 |  |
|  | PhysicalSciences | .076 | .045 | .178 | 1.669 | .097 | -.014 | .166 | .339 | .141 | .114 | .409 | 2.445 |  |
| a. Dependent Variable: YOS1 | | | | | | | | | | | | | | |
